# Peroxidized Linoleic Acid, 13-HPODE, Alters Gene Expression Profile in Intestinal Epithelial Cells

**DOI:** 10.3390/foods10020314

**Published:** 2021-02-03

**Authors:** Nisreen Faizo, Chandrakala Aluganti Narasimhulu, Anna Forsman, Shibu Yooseph, Sampath Parthasarathy

**Affiliations:** 1 Burnett School of Biomedical Sciences, Genomics and Bioinformatics Cluster, College of Medicine, University of Central Florida, Orlando, FL 32816, USA; nfaizo@knights.ucf.edu; 2 Burnett School of Biomedical Sciences, College of Medicine, University of Central Florida, Orlando, FL 32816, USA; Chandrakala.AlugantiNarasimhulu@ucf.edu (C.A.N.); spartha@ucf.edu (S.P.); 3 Department of Biology, Genomics and Bioinformatics Cluster, University of Central Florida, Orlando, FL 32816, USA; Anna.Forsman@ucf.edu; 4 Department of Computer Science, Genomics and Bioinformatics Cluster, University of Central Florida, Orlando, FL 32816, USA

**Keywords:** lipid peroxidation, gene expression, metabolism

## Abstract

Lipid peroxides (LOOHs) abound in processed food and have been implicated in the pathology of diverse diseases including gut, cardiovascular, and cancer diseases. Recently, RNA Sequencing (RNA-seq) has been widely used to profile gene expression. To characterize gene expression and pathway dysregulation upon exposure to peroxidized linoleic acid, we incubated intestinal epithelial cells (Caco-2) with 100 µM of 13-hydroperoxyoctadecadienoic acid (13-HPODE) or linoleic acid (LA) for 24 h. Total RNA was extracted for library preparation and Illumina HiSeq sequencing. We identified 3094 differentially expressed genes (DEGs) in 13-HPODE-treated cells and 2862 DEGs in LA-treated cells relative to untreated cells. We show that 13-HPODE enhanced lipid metabolic pathways, including steroid hormone biosynthesis, PPAR signaling, and bile secretion, which alter lipid uptake and transport. 13-HPODE and LA treatments promoted detoxification mechanisms including cytochrome-P450. Conversely, both treatments suppressed oxidative phosphorylation. We also show that both treatments may promote absorptive cell differentiation and reduce proliferation by suppressing pathways involved in the cell cycle, DNA synthesis/repair and ribosomes, and enhancing focal adhesion. A qRT-PCR analysis of representative DEGs validated the RNA-seq analysis. This study provides insights into mechanisms by which 13-HPODE alters cellular processes and its possible involvement in mitochondrial dysfunction-related disorders and proposes potential therapeutic strategies to treat LOOH-related pathologies.

## 1. Introduction

Dietary lipids, including vegetable oils, contain different quantities of the most common dietary polyunsaturated fatty acid (PUFA), linoleic acid (LA), in the form of triglycerides, which are hydrolyzed by bile and lipases. This process releases large amounts of free fatty acids (FFAs), often in millimolar quantities, that are absorbed by intestinal cells [1]. Depending on processing (e.g., deep frying), the ingested lipids may contain varying quantities of peroxidized PUFAs and their decomposition products [2]. Dietary lipid peroxides (LOOHs) are broken down in the gut, resulting in the production of other lipid peroxidation products, such as epoxy ketones, and the release of peroxidized fatty acids (FAs) that reach the enterocytes, where they get absorbed [3]. It has been demonstrated that dietary LOOHs from overheated oils contribute to the presence of peroxidized FAs in the lipoproteins [4], which indicates that even though dietary LOOHs undergo a set of enzymatic digestion, peroxidized FAs reach the intestine and get absorbed.

LOOHs have been strongly linked to disorders of the digestive system, including inflammatory bowel disease (IBD) and malignancy [5]. Studies have demonstrated that while LOOH is efficiently absorbed by the fully differentiated (Dif) intestinal epithelial cells and transported in the lymph, it is poorly taken up by undifferentiated cells [3,6]. In addition, it has been demonstrated that the uptake of oxidized FAs by Dif Caco-2 cells is dependent on the presence of brush borders and is comparable to the uptake of unoxidized FAs in Dif Caco-2 cells [3]. 13-hydroperoxyoctadecadienoic acid (13-HPODE) is decomposed by cells rapidly into aldehydes, including 4-hydroxynonenal (4-HNE) and oxononanoic acid (ONA), which are cytotoxic and cause the generation of reactive oxygen species (ROS). Previous studies have shown that LOOH caused oxidative stress and the loss of cellular integrity in the intestinal epithelium [7]. Peroxidized fat consumption has also been reported to cause pro-inflammatory changes in the intestine [8,9]. Iron/ascorbate-mediated production of LOOH in Caco-2 cells has been shown to result in activation of NF-κB, which regulates inflammatory processes [10]. Dietary LOOHs have also been implicated in cardiovascular disease [3,11]. Previous studies also showed that peroxidized fat was carried in the chylomicrons [12], which was correlated with the peroxidized fat content in the diet [13]—these are believed to be re-packaged and distributed in the lipoproteins. The presence of peroxidized fat in the chylomicrons has been shown to increase the atherogenicity of dietary cholesterol [14] and promote the absorption of cholesterol [15].

The Caco-2 cell line is a human intestinal epithelial cell line that is derived from human colorectal adenocarcinoma [16]. Under certain cultivation conditions, Caco-2 cells differentiate into a cell monolayer that possesses absorptive features and brush borders resembling human enterocytes. This cell line has been widely used in studies involving cellular uptake, transport, and metabolism of drugs and food molecules, including lipids [3,15,17]. Using the Caco-2 cell line to study lipid transport and metabolism by intestinal epithelium is less challenging than using in vivo animal models [18]. Previous research has revealed that sub-cytotoxic levels of LOOH cause significant injury and mitogenic changes to Caco-2 cells, whereas higher concentrations of LOOH promote cell death [19,20]. LOOHs have also been suggested to induce redox imbalance and disruption of intestinal epithelial turnover [21]. Accordingly, the response of Caco-2 cells to LOOH depends on the amount of LOOH to which intestinal cells are exposed. In addition, LOOH reduced cell membrane fluidity and increased permeability in intestinal epithelial cells [22]; the latter of which is a reported effect in patients with IBD [23]. Changes in membrane fluidity and dynamics could affect several cellular processes [24], including lipid absorption by enterocytes and secretion into lacteals [25]. In addition, LOOH appeared to induce DNA damage as well [7].

A recently published study demonstrated similar results between Caco-2 cells treated with 13-HPODE and mice fed with 13-HPODE [26], which makes treatment of Caco-2 cells with 13-HPODE a good model of dietary LOOH intake. Thus, in this study, we used Caco-2 cells to investigate the effects of 13-HPODE, the most common dietary lipid peroxide, on the metabolic processes, cellular pathways, and phenotype of the intestinal epithelium. We generated gene expression profiles using RNA-seq to gain insights into how Caco-2 cells respond to 13-HPODE. We used these data to identify molecular mechanisms that may explain the contribution of lipid peroxidation to health conditions and their potential role in gut pathology. Here, we conducted RNA sequencing of Caco-2 cells treated with a specific LOOH, 13-HPODE, a lipoxidase-derived product from LA, since LA is the most abundant dietary PUFA [27]. Bioinformatic analyses of the generated RNA-seq data provided valuable information on dysregulated genes and disrupted pathways in Caco-2 cells in response to 13-HPODE, the most common dietary LOOH. We compared the results obtained from 13-HPODE-treated cells with the gene expression profile of Caco-2 cells treated with LA, which represented non-peroxidized lipids. Treatment of Caco-2 cells with LA mimics the intake of vegetable oils such as soybean and canola oils and nuts as LA is the most common omega-6 PUFA in vegetable oils and in the Western diet [28]. In addition, comparable results were seen when intestinal culture cells were treated with pure linoleic acid or lipase-digested sesame oil [29]. Validation of RNA-seq results was carried out using qRT-PCR analysis, which showed consistent results with our RNA-seq data. In future works, these investigations could provide potential therapeutic strategies to treat diseases associated with the consumption of peroxidized linoleic acid.

## 2. Materials and Methods

### 2.1. Cell Culture

Caco-2 cells were purchased from American Type Culture Collection (ATCC) (Rockville, MD, USA). Cells were cultured in Dulbecco’s modified Eagle’s medium (DMEM; Invitrogen, Carlsbad, USA) supplemented with 15% fetal bovine serum (FBS; Invitrogen, Carlsbad, USA), 2 mM L-glutamine (Invitrogen, Carlsbad, CA, USA), and 1% penicillin-streptomycin (Invitrogen, Carlsbad, CA, USA). After attaining confluence, cells were cultured in the same medium supplemented with 7.5% FBS and the same concentration of other constituents. Confluent cells were trypsinized using 0.25% Trypsin-EDTA solution (Thermo Fisher Scientific, Waltham, MA, USA). Caco-2 cells were seeded in 6-well plates and experiments were carried out on fully differentiated cells (Dif; Day-14).

### 2.2. Preparation of Lipid Peroxide 

Stock solution of linoleic acid (LA) (Sigma #W338001-25G, St. Louis, MO, USA) was prepared in ethanol, and LA (200 μM) in phosphate-buffered saline (PBS; Invitrogen, Carlsbad, CA, USA) was prepared for LA treatment of cells. 13-hydroperoxyoctadecadienoic acid (13-HPODE) was freshly prepared in PBS as previously described [9,14,30]. Briefly, LA (200 μM) in PBS (pH 7.4) was oxidized with the addition of 10 U soybean lipoxygenase (Sigma #L6632-1MU), which can be easily miscible with the medium. Conjugated diene formation during oxidation was monitored by scanning the absorption between 200 nm and 300 nm using a spectrophotometer (Uvikon XL, Biotek Instruments, El Cajon, CA, USA), using PBS as the reference. The conversion of linoleic acid into its oxidized form was observed as an increase in the optical density at 234 nm. Peroxide content was determined using a leucomethylene blue (LMB) assay [31]. The whole solution of the freshly prepared 13-HPODE was filter-sterilized to reduce the risk of contamination and used for the cell culture experiments within 2 h of preparation to minimize spontaneous peroxide decomposition.

### 2.3. Treatment of Cells with 13-HPODE/LA

As previously described [26], Dif Caco-2 cells were starved in serum-free medium for 3 h prior to treatment. Cells were treated with 100 µM of either 13-HPODE or LA for 24 h. Untreated control cells were maintained with equal amounts of PBS to match the treatments. After 24 h incubation, cells were rinsed with PBS and harvested into TRIzol for total RNA isolation. Experiments were run in triplicate.

### 2.4. Total RNA Extraction and Quantification

Cells were lysed directly in the 6-well culture plates using TRIzol reagent (Invitrogen, 15596026). Cell lysate was transferred to tubes and chloroform was added. The samples were vortexed and incubated at room temperature for 3 min then centrifuged at 12,400× *g* for 15 min. The upper aqueous phase, containing RNA, was transferred into fresh tubes. Isopropyl alcohol was added to the samples and centrifuged for 10 min at 12,000× *g* to precipitate RNA from the aqueous phase. Total RNA was washed with ethanol at 7500× *g* for 5 min and air-dried for 2–3 min. RNA was resuspended in RNase-free water, then sample concentration, purity, and quality were determined using a NanoDrop spectrophotometer (Thermo Fisher Scientific, Waltham, MA, USA), which showed absorbance ratios of 1.8–2 at 260 nm and 280 nm. Any co-extracted DNA was removed from RNA samples using the TURBO DNA-free kit (Invitrogen, AM1907, Carlsbad, CA, USA), following the manufacturer’s instructions.

### 2.5. RNA-seq Library Preparation and Sequencing

We isolated mRNA from total RNA samples using the NEBNext Poly(A) mRNA Magnetic Isolation Module (New England Biolabs, E7490S, Ipswich, MA, USA) with an approximate input of 500 ng of total RNA per sample. RNA-seq libraries were prepared from mRNA samples using the NEBNext Ultra II Directional RNA Library Prep kit for Illumina (New England Biolabs, E7760S) according to the manufacturer’s instructions, including NEBNext Sample Purification Bead cleanups to remove unincorporated primers and adapters. Samples were fragmented for 15 min at 94 °C to achieve a target fragment size of 200 base pairs (bp). Indexed adaptors for Illumina sequencing (New England Biolabs, E7710, E7730) were ligated to libraries through 8 cycles of PCR. Library quality was assessed using High Sensitivity D1000 reagents (Agilent Technologies, 5067-5585, Santa Clara, CA, USA) on a TapeStation 2200 instrument (Agilent Technologies, Santa Clara, CA, USA). Library concentrations were determined using the NEBNext Library Quant Kit for Illumina (New England Biolabs, E7630S) following the manufacturer’s instructions. Three dilutions of each library were prepared (1:1000, 1:10,000, 1:100,000) and plated in triplicate on a 96-well qPCR plate along with manufacturer-supplied standards (20 µL reactions). Concentration data were used to ensure equimolar pooling across libraries for multiplexing. The final library pool was checked for quality using the High Sensitivity D1000 ScreenTape assay, which showed good quality with a maximum peak size of 337 bp (Figure S1), and sent to GENEWIZ (South Plainfield, NJ, USA) for sequencing (HiSeq4000 2 × 150 bp). The number of sequencing reads ranged between 48 and 79 million reads with a mean quality score > 37.

### 2.6. Sequence Data Processing

The following pipeline was used to analyze the paired-end sequencing reads. We used FastQC (version v0.11.7) to check the quality of reads, which were all of good quality scores > 30, and the presence of adapters or overrepresented sequences [32]. Trimmomatic (version 0.36) was used to remove adapters and poor-quality bases [33]. Hisat2 (version 2.1.0) was used to align the reads to a human reference genome (Ensembl/Genome Reference Consortium Human Build 38, GRCh38) [34]; all samples showed overall read alignment rates > 90%. Then, FeatureCounts (version 1.5.0) was run to count the number of fragments mapped to a specific gene/exon [35]. We used DESeq2 (version 1.30.0), an R-package which uses negative binomial distribution to model read counts, to identify differentially expressed genes (DEGs; adjusted *p* < 0.05) between different groups [36]. Gene symbols were used from the Ensembl database.

### 2.7. Enrichment Analyses

Differentially expressed genes were evaluated further using gene ontology and enrichment analyses using the enrichGO function of the clusterProfiler (version 3.18.0) R package [37], and Generally Applicable Gene-set Enrichment (GAGE, version 2.40.0; also R package) [38], respectively. Gene ontology (GO) analysis was carried out by running the enrichGO function on the list of DEGs (adjusted *p* < 0.05) (from DESeq2) in the treated cell group to identify enriched GO terms including biological processes, molecular functions, and cellular components. Pathway and gene set enrichment analyses (GSEA) were conducted by running the gage function on the list of DEGs and their log2 fold change scores. This generated a list of dysregulated Kyoto Encyclopedia of Genes and Genomes (KEGG) pathways, their *p* values, and their direction (downregulated or upregulated).

### 2.8. Validation via qRT-PCR

RNA-seq results were technically confirmed via qRT-PCR using the same RNA samples used for the RNA-seq study. Fourteen representative DEGs were chosen to perform qRT-PCR for 13-HPODE-treated cells and eleven DEGs for LA-treated cells. SsoAdvanced^TM^ Universal SYBR^®^ Green Supermix (BioRad, 1725271), 2.5 μM of primer (forward and reverse each), and 0.1 ng of cDNA was used in a 10 μL PCR reaction. The protocol was set as 95 °C for 30 s (initial denaturation), and 40 cycles of 95 °C for 15 s (denaturation) and 60 °C for 30 s (annealing/extension). *GAPDH* was used as a housekeeping gene. Relative mRNA expression levels were determined using the ΔΔ Ct method. A *t*-test was used to determine the statistical differences between the treated and untreated groups and the data were presented as means ± SD. Primer sequences are provided in Table S1.

## 3. Results

We incubated Dif Caco-2 cells with 100 µM of 13-HPODE or LA for 24 h. We chose these concentrations as the proximal intestine is exposed to millimolar concentrations of FA and our preliminary results showed little or no cytotoxicity [3,15]. The control (untreated) group was maintained in PBS. Experiments were run in triplicate (details are explained in the Section 2). Following RNA extraction, processing, and sequencing, differential gene expression and enrichment analyses were carried out. Principal component analysis showed that replicate samples demonstrated similarity in gene expression with respect to treatment, and there was good separation between untreated and treated groups (Figure S2). The log ratio vs. mean average (MA) plots (Figure S3) show that differentially expressed genes (DEGs; red dots) with a large mean expression across untreated and treated cell groups call for significance.

### 3.1. 13-HPODE-Treated Caco-2 Cells

#### 3.1.1. Differential Gene Expression

**Figure 1:**
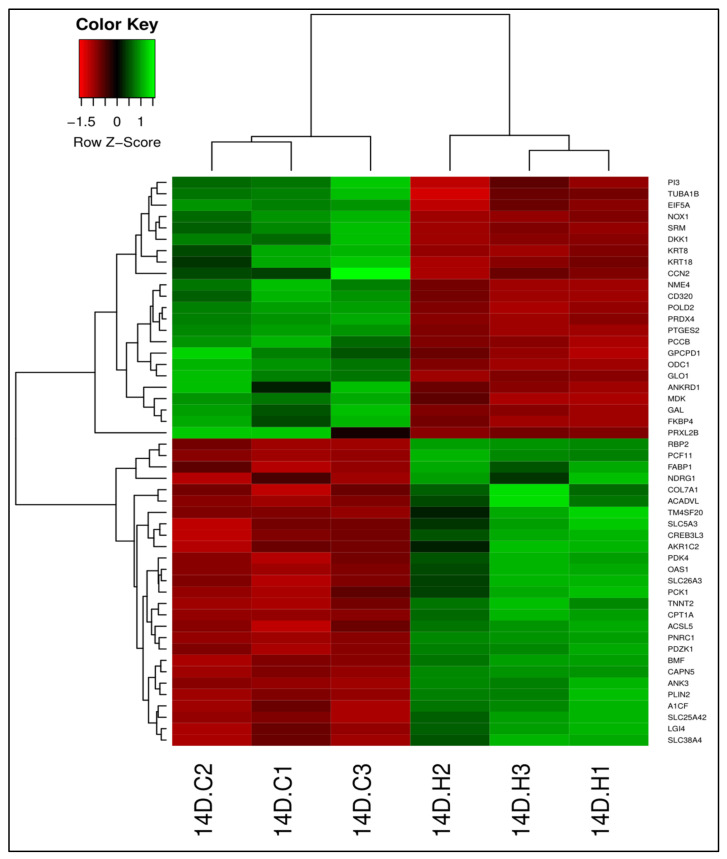
Differential gene expression between 13-HPODE-treated cells and untreated control cells. Heatmap shows the top 50 differentially expressed genes (DEGs; adjusted *p* < 0.05) in 13-HPODE-treated Caco-2 cells (14D.H1, 14D.H2, and 14D.H3) compared to untreated control cells (14D.C1, 14D.C2, and 14D.C3). Green, upregulated; red, downregulated.

Using DESeq2, we identified 3094 DEGs (adjusted *p* < 0.05) between 13-HPODE-treated cells and untreated cells (Table S2); 1692 genes were downregulated and 1402 genes were upregulated in 13-HPODE-treated cells relative to untreated cells. Among the upregulated genes were genes involved in lipid metabolism such as *PLIN2*, *FABP1*, *CPT1A*, and *PCK1*, which are involved in PPAR signaling, which on one hand has shown to exert anti-inflammatory effects in obesity, diabetes, and cardiovascular disease [39], but on the other hand, could have both anti-proliferative and carcinogenic effects [40]. *CPT1A* and *ACADVL*, which are involved in mitochondrial beta-oxidation of fatty acids, and *PDK4* and *PCK1*, which have a role in lipid and glucose metabolism, were upregulated. Induction of these genes may promote both fat metabolism and gluconeogenesis. Genes involved in stress response, such as *CREB3L3* and *NDRG1*, were also induced. Although we observed reduced expression of glutathione peroxidases *GPX1* and *GPX7*, there was upregulation of *GCLC*, which is essential for glutathione (GSH) synthesis, which might indicate increased GSH contents. Reduced glutathione peroxidase activity has been linked with colon cancer [41], cardiovascular disease [42], obesity, and insulin resistance [43]. We also observed upregulation of some nuclear factor erythroid 2-related factor 2 (Nrf2) target genes that play a role in the antioxidant defense systems such as *HMOX1, CAT, UGT2B4*, and *TXNRD1*. Several solute carrier (SLC) transporters were upregulated, such as *SLC26A3*, a chloride transporter; *SLC38A4*, an amino acid transporter; and *SLC5A3*, a myo-inositol transporter, indicating changes in substrate transport across the cell membrane. In addition, SLC transporters have been implicated in various diseases, including IBD and metabolic diseases [44,45]. Other upregulated genes included *COL7A1*, which forms fibrils between epithelial cell basement membrane and extracellular matrix, and *PDZK1*, which regulates epithelial cell surface proteins and is involved in cholesterol metabolism. Among the downregulated genes were *ODC1* and *POLD2*, which are important for polyamine synthesis and DNA replication, respectively. *GPCPD1*, which is involved in glycerophospholipid biosynthesis, and *PTGES2*, which is involved in prostaglandin E synthesis, were also downregulated. 13-HPODE treatment also caused the reduction of *NOX1*, a NADPH oxidase that generates ROS, as well as *DKK1*, which is an inhibitor of Wnt signaling [46]. Figure 1 shows the top 50 DEGs in 13-HPODE-treated Caco-2 cells compared to untreated cells.

#### 3.1.2. Gene Ontology

**Figure 2:**
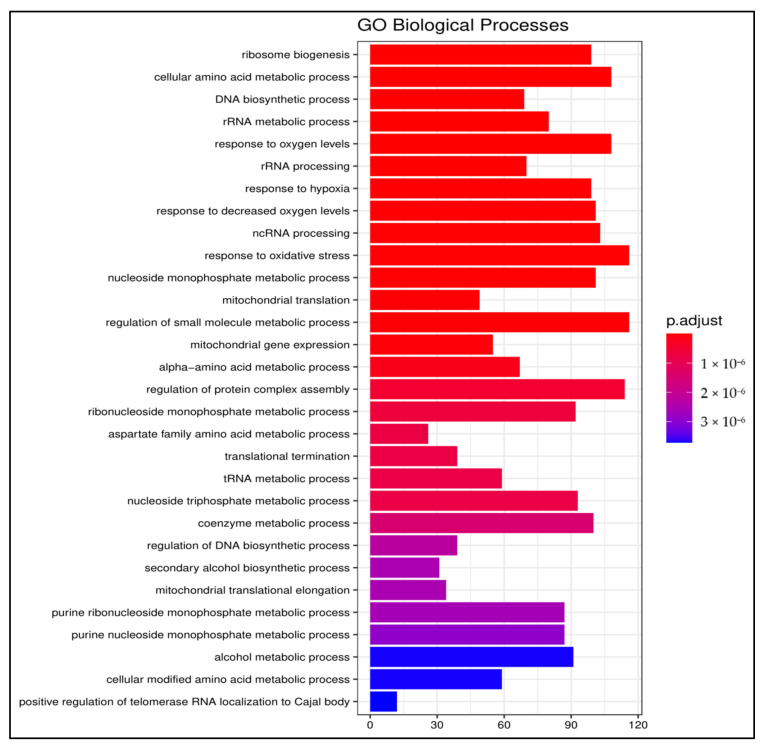
Biological process enrichment upon treating Caco-2 cells with 13-HPODE. Top enriched gene ontology (GO) biological processes in 13-HPODE-treated differentiated Caco-2 cells relative to untreated control cells (adjusted *p* < 0.05).

Gene ontology analysis was performed using the enrichGO function of the clusterProfiler R-package. The results revealed the enrichment of diverse biological processes (adjusted *p* < 0.05) involved in translation, ribosome biogenesis, RNA processing, response to hypoxia and oxidative stress, mitochondrial translation, and gene expression. Purine nucleoside monophosphate, alcohol, and amino acid metabolic processes (Figure 2), as well as carbohydrate and lipid metabolic processes (not shown), were also enriched due to 13-HPODE treatment.

Among the enriched molecular functions (adjusted *p* < 0.05) in Caco-2 cells treated with 13-HPODE were ATPase, oxidoreductase, antioxidant, electron transfer, RNA polymerase I, and helicase activities. Additionally, coenzyme, carboxylic acid, heat shock protein, and cadherin and chaperone binding functions were enriched (Figure S4).

The enriched cellular components (adjusted *p* < 0.05) included the mitochondrial inner membrane, matrix, ribosome, and protein complex, as well as ribosomal subunits, the spliceosomal complex, focal adhesion, and the cell-substrate adherens junction (Figure S5). The brush border and apical plasma membrane were also enriched (not shown).

#### 3.1.3. Pathway Enrichment Analysis 

We used the gage R-package to perform pathway enrichment analysis (Table 1). Among the top upregulated KEGG pathways (*p* < 0.05) in 13-HPODE-treated cells, relative to untreated cells, were steroid hormone biosynthesis, bile secretion, and protein digestion and absorption. We also observed upregulation of importantly relevant pathways such as metabolism of xenobiotics by cytochrome P450, focal adhesion, and PPAR signaling, but with slightly higher *p* values.

Among the downregulated pathways (*p* < 0.05) observed for 13-HPODE-treated cells, relative to untreated cells, were spliceosome, RNA transport, ribosome biogenesis, and RNA polymerase pathways, as well as purine and pyrimidine metabolism. Oxidative stress can cause base modification and DNA damage [47]. Pathways of cell cycle, DNA replication, and excision repair appeared to be suppressed as well. The proteasomal pathway involved in proteolytic degradation of intracellular protein was also downregulated. The results also showed a reduction in the oxidative phosphorylation pathway.

### 3.2. LA-Treated Caco-2 Cells

#### 3.2.1. Differential Gene Expression

**Figure 3:**
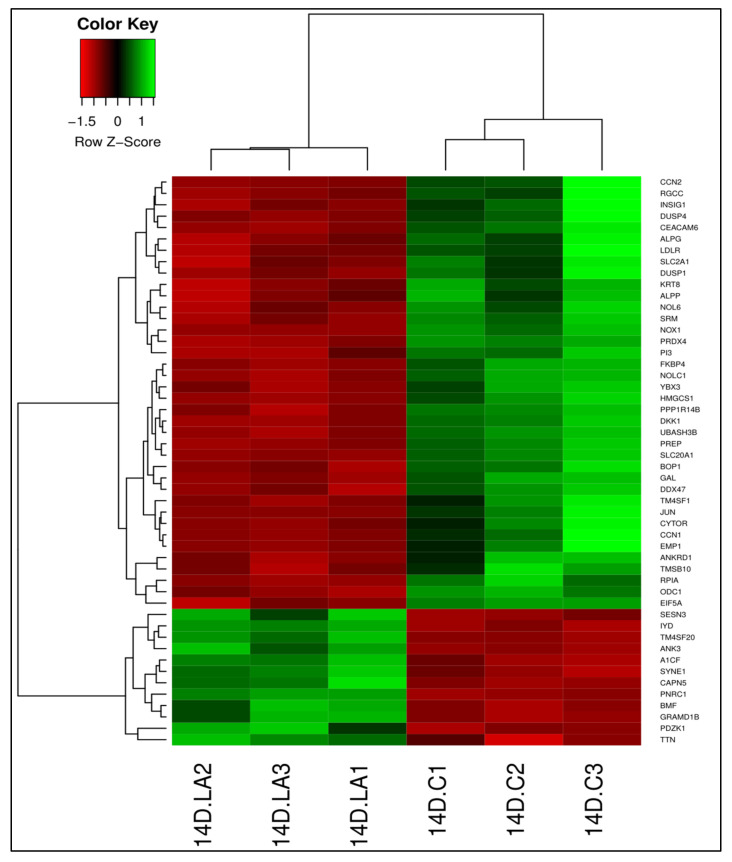
Differential gene expression between linoleic acid (LA)-treated cells and untreated control cells. Heatmap shows the top 50 DEGs (adjusted *p* < 0.05) in LA-treated Caco-2 cells (14D.LA1, 14D.LA2, and 14D.LA3) compared to untreated cells (14D.C1, 14D.C2, and 14D.C3). Green, upregulated; red, downregulated.

We identified 2862 DEGs (adjusted *p* < 0.05) in LA-treated cells compared to untreated cells (Table S3); 1179 genes were upregulated and 1683 genes were downregulated in LA-treated cells. As in 13-HPODE-treated cells, *PDZK1* was induced. *TTN*, which has been reported as a key component of intestinal epithelial brush borders [48], was also upregulated. Among the downregulated genes in LA-treated cells were *RGCC*, a cell cycle regulator; *CYTOR*, a long non-coding RNA that enhances proliferation; and *CEACAM6*, which is a cell adhesion molecule that promotes tumor progression. *HMGCS1*, involved in cholesterol biosynthesis; *DDX47*, involved in spliceosome and ribosomal RNA processing; *EIF5A*, a translation elongation factor; and *ODC1* were suppressed as well. Reduction in the expression of these genes may indicate reduced cell proliferation upon LA treatment. Genes involved in the regulation of metabolic pathways were also downregulated, such as *INSIG1*, which regulates lipid synthesis and glucose homeostasis, and the *RPIA* gene, involved in carbohydrate metabolism. In addition, genes that are involved in the regulation of cellular fate and processes were suppressed, such as *JUN*, a transcription factor that regulates gene expression; *DUSP4*, dual specificity phosphatase of mitogen-activated protein kinase; and *DKK1*. Other downregulated genes included *PI3*, a peptidase inhibitor (antimicrobial); *SLC2A1*, a glucose transporter; and *SLC20A1*, a phosphate transporter [46]. Figure 3 illustrates the top 50 DEGs in LA-treated Dif Caco-2 cells compared to untreated cells.

#### 3.2.2. Gene Ontology

**Figure 4:**
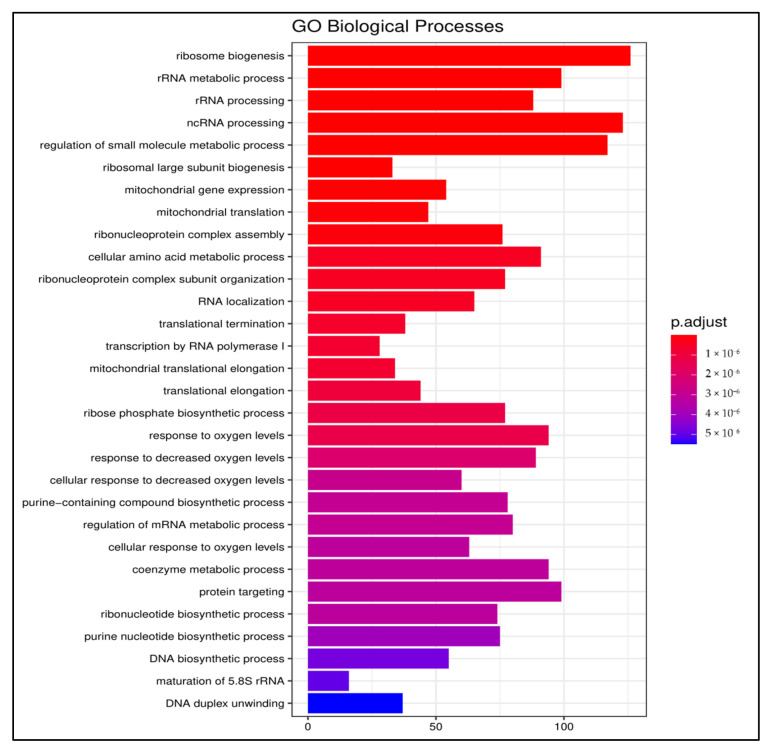
Biological process enrichment upon treating Caco-2 cells with LA. Top enriched GO biological processes in LA-treated differentiated Caco-2 cells relative to untreated cells (adjusted *p* < 0.05).

Among the enriched biological processes in LA-treated cells relative to untreated cells were ribosome biogenesis; rRNA and ncRNA processing; small molecule, amino acid, and coenzyme metabolic processes; ribonucleoprotein complex assembly; and mitochondrial translation and gene expression. In addition, the response to decreased oxygen levels, purine nucleotide biosynthesis, DNA duplex unwinding, anion transport, and carbohydrate metabolic processes were enriched in LA-treated cells (Figure 4).

Molecular functions enriched in LA-treated Dif Caco-2 cells included organic acid transmembrane transporter activity, helicase, ligase, RNA polymerase, and ATPase activities. Moreover, ribonucleoprotein complex, cadherin, ATPase, coenzyme, organic acid, cell adhesion molecule, and RNA binding were also enriched (Figure S6).

Among the enriched cellular components in LA-treated cells relative to untreated cells were preribosome, and organellar and mitochondrial ribosomes. Mitochondrial outer/inner membrane and matrix, cellular and organellar outer membrane, and envelop lumen components were also enriched. In addition, complexes such as proteasome, spliceosome, and endopeptidase complexes were enriched in LA-treated cells, as well as focal adhesion and adherens junctions (Figure S7). The brush border and apical plasma membrane were also enriched (not shown).

#### 3.2.3. Pathway Enrichment Analysis

Linoleic acid treatment of Dif Caco-2 caused upregulation (*p* < 0.05) of metabolic processes including retinol, xenobiotic, and drug metabolism by cytochrome P450, relative to untreated cells. The protein digestion and absorption pathway was also upregulated.

Among the pathways downregulated (*p* < 0.05) in LA-treated cells, relative to untreated cells, were ribosome biogenesis, spliceosome, RNA transport, and RNA polymerase pathways. As in 13-HPODE-treated cells, purine and pyrimidine metabolism, oxidative phosphorylation, proteasome, cell cycle, and excision repair pathways were also suppressed in LA-treated cells. Toll-like receptor signaling, which activates innate immunity, was downregulated (Table 2).

### 3.3. Differential Gene Expression between 13-HPODE-Treated and LA-Treated Cells 

We identified 291 DEGs (adjusted *p* < 0.05) between 13-HPODE-treated and LA-treated cells (Figure S8; Table S4). We found that genes involved in PPAR signaling showed higher expression levels in 13-HPODE-treated cells compared to LA-treated cells. Among those genes were CPT1A, which is involved in FA oxidation; PLIN2, which coats lipid droplets; ACSL5 and FABP1, which are involved in FA transport; HMGCS2, which is involved in ketogenesis; and PCK1. Upregulation of the latter gene and PDK4 could promote gluconeogenesis, as mentioned earlier. These results might suggest that 13-HPODE is a more potent activator of PPAR signaling than LA, although both oxidized and unoxidized LA have been shown in previous studies to activate PPARs [30,49]. This also indicates a significant effect of 13-HPODE on glucose and lipid metabolism and transport as PPAR signaling has been shown to regulate metabolic processes including FA and glucose metabolism, as well as cell proliferation and differentiation [50,51]. Additional genes involved in FA oxidation, including SLC25A20 and ACADVL, were upregulated in 13-HPODE-treated cells compared to LA-treated cells. On the other hand, G6PD, which plays a role in FA and cholesterol biosynthesis, also showed higher expression in 13-HPODE-treated cells relative to LA-treated cells. Lower expression of APOH, which has an atheroprotective role by inhibiting the uptake of oxidized low density lipoprotein (LDL) [52], was observed in 13-HPODE-treated cells relative to LA-treated cells. We observed increases in the expression of genes involved in defense mechanisms in 13-HPODE-treated relative to LA-treated cells. Among these were CREB3L3; ABCG2, which is a xenobiotic transporter that extrudes toxins from cells; and CYP2B6 and AKR1B1, which metabolize xenobiotics and aldehydes.

We observed increased expression of genes that play a role in detoxification in LA-treated cells relative to 13-HPODE-treated cells. Among these were EPHX2, which is involved in xenobiotic metabolism; ADH6, which metabolizes alcohols and lipid peroxidation products; and ALDH6A1, which plays a role in protective detoxification of aldehydes. Sucrase-isomaltase gene, SI, which is involved in dietary carbohydrate digestion and is expressed in the intestinal brush border, showed upregulated expression in LA-treated cells relative to 13-HPODE-treated cells. Interestingly, cell adhesion molecules CEACAM1/M6/M5, which are considered biomarkers of tumor progression and metastasis, showed low expression levels in LA-treated cells relative to 13-HPODE-treated cells.

### 3.4. Validation of RNA-Seq Results

**Figure 5:**
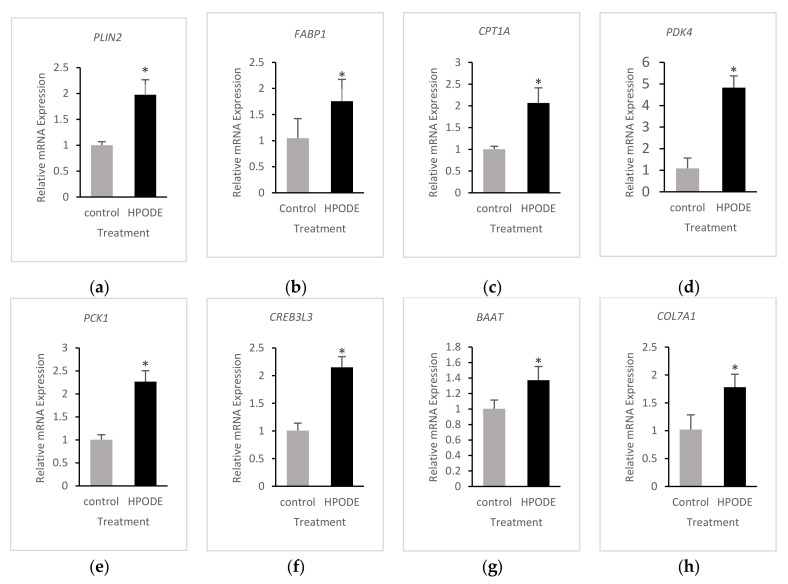

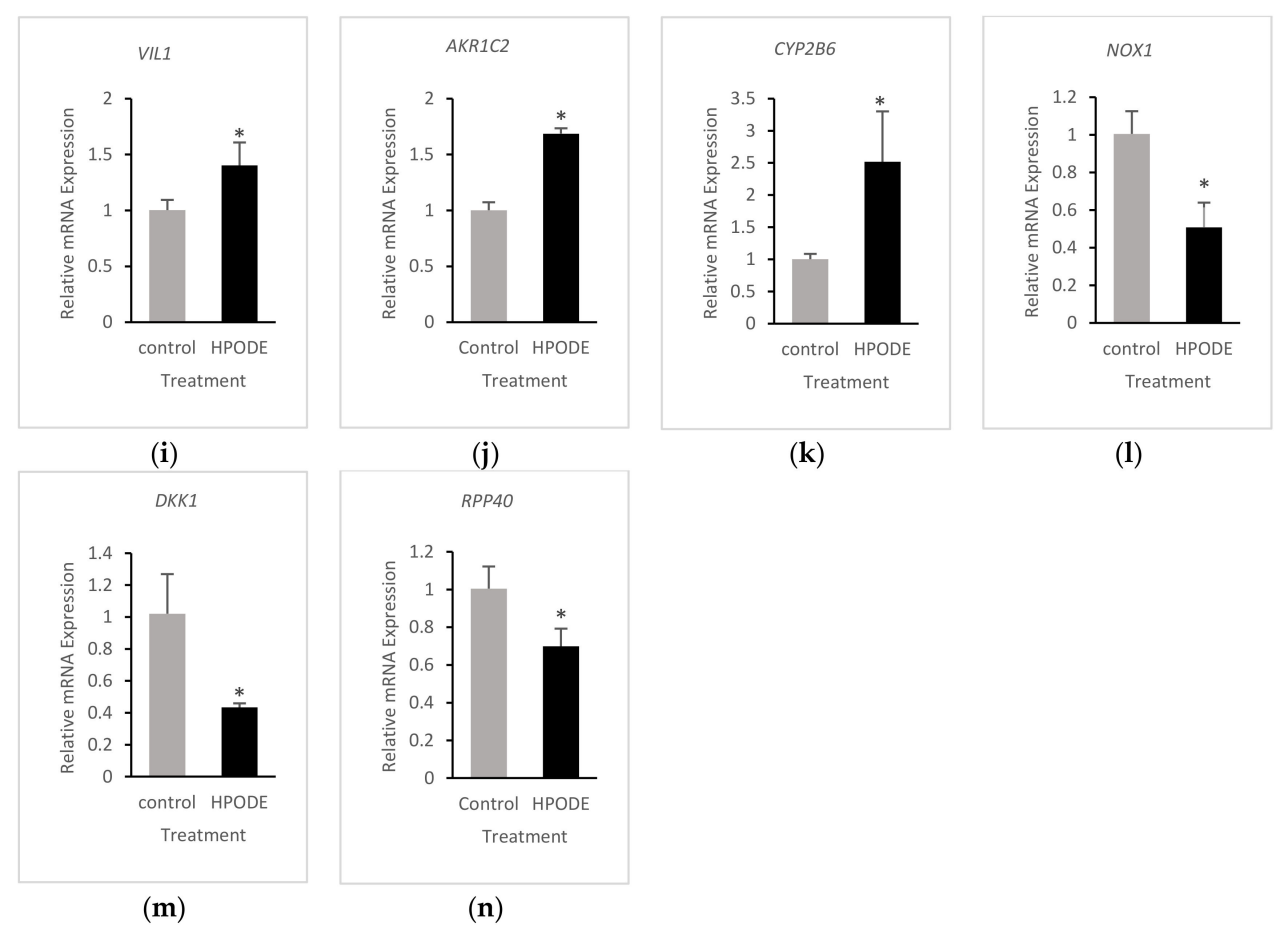
Quantitative Real-time PCR validation of RNA-seq results in 13-HPODE-treated cells (**a**–**n**). qRT-PCR of representative DEGs from RNA-seq data. Relative mRNA expression of genes is presented in 13-HPODE-treated (HPODE) Caco-2 cells with statistical significance of * *p* < 0.05 compared to untreated (control) cells. Results were normalized to GAPDH.

RNA-seq results were verified via qRT-PCR. Different gene sets were selected for validation in each treatment according to the differential gene expression and pathway analysis in the treated groups relative to the untreated group. Fourteen representative DEGs were chosen to perform qRT-PCR for 13-HPODE-treated cells and eleven DEGs for LA-treated cells. Genes involved in PPAR signaling, as well as mitochondrial beta oxidation, such as *PLIN2*, *FABP1*, and *CPT1A*, were upregulated in 13-HPODE-treated cells relative to untreated cells (Figure 5a,c). *PDK4* and *PCK1* genes, which play a role in the regulation of lipid and glucose metabolism, were also upregulated in 13-HPODE-treated cells, suggesting enhanced gluconeogenesis in these cells relative to untreated cells (Figure 5d,e). *CREB3L3*, inflammatory response gene, and *BAAT*, involved in bile acid synthesis, were upregulated by 13-HPODE compared to untreated cells (Figure 5f,g). 13-HPODE treatment was associated with increased expression of *COL7A1*, a collagen type VII alpha 1 chain, and *VIL1* genes (Figure 5h,i), involved in focal adhesion and the brush border cytoskeleton, respectively, which might indicate enhanced cell differentiation. Aldo/keto reductase *AKR1C2*, which is involved in steroid hormone and bile acid synthesis, was induced by 13-HPODE (Figure 5j). *CYP2B6* was also upregulated in 13-HPODE-treated cells (Figure 5k). 13-HPODE reduced the expression of NADPH oxidase *NOX1* (Figure 5l). Other genes with reduced expression when treated with 13-HPODE treatment included *DKK1* and *RPP40*, which are involved in Wnt signaling and rRNA processing, respectively (Figure 5m,n).

**Figure 6:**
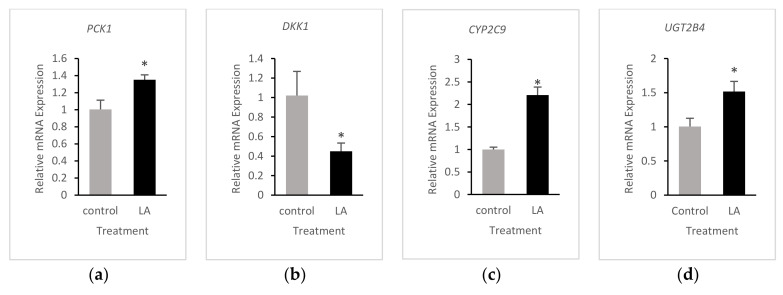

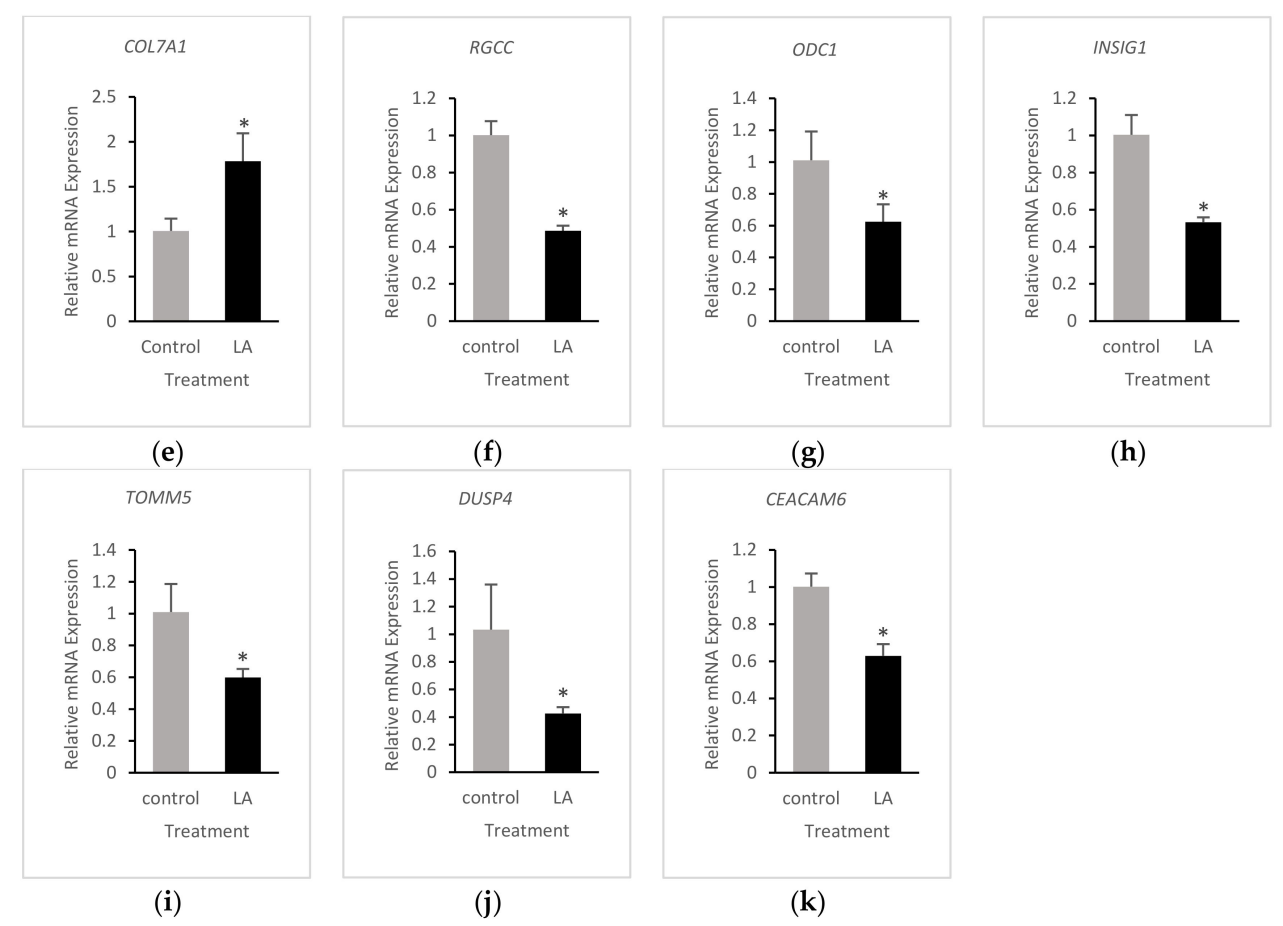
Quantitative Real-time PCR validation of RNA-seq results in LA-treated cells (**a**–**k**). qRT-PCR of representative DEGs from RNA-seq data. Relative mRNA expression of genes is presented in LA-treated (LA) Caco-2 cells with statistical significance of * *p* < 0.05 compared to untreated (control) cells. Results were normalized to GAPDH.

The relative expression of *PCK1* and *DKK1* in LA-treated cells was similar to that of 13-HPODE-treated cells when both were compared to untreated cells (Figure 6a,b). In addition, *CYP2C9*, a cytochrome P450 mono-oxygenase; *UGT2B4*, a detoxifying UDP glucuronosyltransferase; and *COL7A1* were upregulated in LA-treated cells relative to untreated cells (Figure 6c–e). Reduced expression of *RGCC* and *ODC1* could be attributable to reduced proliferative potential and enhanced differentiation (Figure 6f,g). LA treatment led to downregulation of *INSIG1* and *TOMM5* genes, which might affect metabolic processes and mitochondrial function (Figure 6h,i). *DUSP4*, as well as cell adhesion molecule *CEACAM6*, was reduced in LA-treated cells as well, which could be a protective response against tumor progression (Figure 6j,k). These results are consistent with the RNA-seq data.

## 4. Discussion

LA, an essential PUFA consumed through the human diet, is an important cellular component. It is a precursor of lipid peroxides including hydroxyoctadecadienoic acid (HODE), the reduced form of 13-HPODE [53]. LOOHs have been linked to several pathological conditions including cardiovascular disease, degenerative disease, and malignancy [54]. Diet is a major source of LOOH, and dietary peroxides are absorbed, transported, and incorporated into lipoproteins that carry these lipids into cells and tissues. We used mRNA sequencing to investigate how the most common dietary LOOH, 13-HPODE, modulates the gene expression profile in Dif Caco-2 cells, the results of which could provide insights into changes in the physiological processes of cells and the mechanisms by which 13-HPODE may contribute to disease. Treating Caco-2 cells with 13-HPODE, a peroxidized LA, significantly alters metabolic and signaling pathways, as well as different cellular processes.

In the current study, we have demonstrated that 13-HPODE increased the expression of aldo/keto reductases, such as *AKR1C2*, that are essential for steroid hormone synthesis. Studies have reported the ability of the intestine to synthesize steroid hormones [55,56]. Steroid hormones have been shown in a previous study to play a role in maintaining the intestinal epithelial barrier [57]; on the other hand, they may not only cause inhibition of T-cell response [55], but may also promote immune function [58], which might contribute to inflammatory disease. It has been reported previously that oxidized LA can induce steroid hormone synthesis in rat and human adrenal cells [59,60]. Upregulated aldo/keto reductases might indicate that 13-HPODE could be converted to aldehydic products in intestinal epithelial cells and promote the detoxification of reactive carbonyl species and the reduction of 4-HNE, which may enhance adaptive response [61]. In addition, oxidized LA metabolites have been linked to obesity and shown to induce synthesis of steroids [62]. Oxidized LA may possess bile acid activity and the ability to form mixed micelles with fat, which increases cholesterol solubilization and absorption [15]. Although bile secretion occurs in the liver, our pathway analysis showed that this pathway was enriched in Caco-2 cells treated with 13-HPODE. We found that the *BAAT* gene, which is responsible for bile acid conjugation that enhances fat solubility, was upregulated in 13-HPODE-treated cells. This could propose another mechanism by which 13-HPODE increases lipid solubilization. A previous study demonstrated that bile secretion in response to a high-fat diet could injure the intestinal epithelium and that secondary bile acids produced by intestinal flora may induce tumor formation [63]; this might be studied as an indirect mechanism by which LOOH could contribute to malignancy. On the other hand, the *BAAT* gene was not differentially expressed in LA-treated cells relative to untreated cells. It is also worth mentioning that both 13-HPODE and LA treatments enhanced the expression of *APOB*, apolipoprotein B, which is a major component of chylomicron and LDL, along with *A1CF*, a complementation factor of ApoB mRNA editing enzyme complex essential for the generation of ApoB48, which promotes dietary fat absorption [64].

Although upregulation of the PPAR signaling pathway upon treating cells with 13-HPODE had a slightly higher *p*-value of 0.1, several downstream genes involved in fatty acid and lipid homeostasis were among the top differentially upregulated genes (adjusted *p* < 0.05) in 13-HPODE-treated cells relative to untreated cells. Among those were *PLIN2*, which forms the lipid droplet coat, and *FABP1*, which may promote cholesterol uptake and protect against oxidative stress caused by LOOH [65]. *HMGCS2*, which is involved in ketogenesis and has been found to promote cellular differentiation of Caco-2 cells [66], was also upregulated. *CPT1A* and *PCK1* were also among the differentially upregulated genes of PPAR signaling which may indicate promoted fatty acid oxidation, as well as gluconeogenesis. As oxidized LA is considered a ligand that activates PPAR signaling, which plays multiple roles ranging between metabolism, anti-inflammatory, and anti- and pro-carcinogenic effects, PPAR signaling should be considered a powerful form of crosstalk between 13-HPODE and peroxidized lipid-related diseases and should be studied further. Moreover, *CREB3L3*, a transcription factor that works with PPARα in an auto-loop manner [67] and is involved in unfolded stress response and lipid metabolism, was upregulated in 13-HPODE-treated cells. Although we observed upregulation of several genes involved in PPAR signaling and FA oxidation, including *PLIN2*, *PCK1*, and *CPT1A* in LA-treated cells, the expression of these genes appeared to be higher in 13-HPODE-treated cells compared to LA-treated cells. This suggests that 13-HPODE may have a more powerful effect on PPAR signaling and FA oxidation than non-peroxidized LA. We also observed higher expression of *G6PD*, required for FA and cholesterol biosynthesis, and a lower expression of atheroprotective *APOH* in 13-HPODE-treated cells compared to LA-treated cells, which could propose a mechanism by which LOOHs contribute to the development of cardiovascular disease.

Our results indicate that 13-HPODE may enhance detoxification by cytochrome P450 enzymes, which play a role in the metabolism of ingested drugs and toxins, and in the synthesis of steroid hormones, bile acids, and some other fats [68,69]. Upregulated *CYP1B1/2C9/2B6* may be a protective response against 13-HPODE treatment as it is considered a harmful metabolite of oxidized LA. in addition to aldo/keto reductases’ function in steroid hormone synthesis, they are also involved in xenobiotic metabolism by cytochrome P450, as well as bile acid synthesis and transport [70,71]. In the current study, LA also induced cytochrome P450 enzymes, including *CYP2C9*. Moreover, LA treatment resulted in higher expression of alcohol and aldehyde dehydrogenases *ADH6* and *ALDH6A1* in LA-treated cells compared to 13-HPODE-treated cells, which could indicate a protective effect of LA against lipid peroxidation products, including aldehydes. This may also suggest that LA could undergo oxidation in the intestinal cells to which the cells respond by enhancing the detoxification mechanism.

As 13-HPODE comes in contact with the cell membrane, we could expect oxidation of vitamin E (Tocopherol; TP) and the formation of tocopheryl quinone (TQ), which is anti-androgenic [72] and has an apoptotic effect that has been reported to inhibit colon cancer cell growth [73]. Moreover, since we observed that 13-HPODE caused downregulation of glutathione peroxidases *GPX1* and *GPX7* and upregulation of the catalytic subunit of glutamate-cysteine ligase, *GCLC*, essential for glutathione (GSH) synthesis, the cells could accumulate GSH. The latter has previously shown to convert the tocopheroxyl radical back to TP to maintain its scavenging effect on reactive oxygen species (ROS) via oxidation of vitamin E, thus preventing further lipid oxidation and inhibiting cell proliferation [74]. TP regeneration has been also linked to thioredoxin reductase [75], which was upregulated in 13-HPODE-treated cells. TP has been shown to reduce chemokines in human aortic endothelial cells (HAECs) [76], and has been shown to reduce expression of the chemokine *CXCL1* [77]. In our study, we observed downregulation of *CXCL1*, *CXCL8* (*IL-8*), and *CCL20* chemokines in 13-HPODE-treated cells. It has been suggested that tocopheryl hydroquinone (TQH) is a more powerful antioxidant than TQ [78,79]. In our study, upregulation of cytochrome p450 oxidoreductase (*POR* gene) [78], which is reported to catalyze the formation of TQH, was observed in 13-HPODE-treated cells. TQ has been shown to induce apoptosis via activation of the caspase 3 cascade and to reduce anti-apoptotic Bcl-2 [80]. In this context, we observed upregulation of *CASP3* and a number of Bcl family members that have apoptotic activity, such as *BMF, BCL2L11*, and *BNIP5* (this gene has not been studied yet), and downregulation of the anti-apoptotic *BAG1* gene in 13-HPODE cells. This is consistent with a recently published study [26] which demonstrated reduced cell viability on annexin V staining of Caco-2 cells treated with 100 μM 13-HPODE.

Although we did not observe expression changes in members of activator protein-1 (AP-1) or *NFE2L2* (Nrf2), we did observe upregulation of some target genes that play a role in the antioxidant defense systems, including *HMOX1, CAT, UGT2B4*, and *TXNRD1*, as well as downregulation of *SOD1* in 13-HPODE-treated cells. This indicates that a degree of the antioxidant protective response was enhanced by 13-HPODE. Moreover, we observed increased expression of *FOXO3* and *FOXO4* transcription factors, by which 13-HPODE could modulate insulin signaling and the antioxidant response and could trigger apoptosis [81]. In addition to *CAT*, another FOXO-regulated gene, *CDKN1A* (p21), which inhibits cell proliferation in response to DNA damage, was also upregulated in 13-HPODE-treated cells.

13-HPODE treatment caused the downregulation of events that have been reported to be reduced during Caco-2 cell differentiation [82]. Among the pathways downregulated due to 13-HPODE treatment were RNA transport, spliceosome, and translation machinery. In parallel with this, events of the cell cycle, DNA replication and repair, and protein degradation, including the proteasomal pathway, were also suppressed. Under culture conditions, Caco-2 cells undergo extensive genetic reprogramming during differentiation and lose their tumorigenic phenotype [16]. This genetic reprogramming includes downregulation of genes involved in cell cycle progression, DNA replication/repair, as well as genes involved in RNA splicing/transport and protein degradation, which indicates reduced proliferation; this was reported by Mariadason et al. [82]. Hence, downregulation of these events upon treating the cells with 13-HPODE or LA, as we observed in the current study, may suggest a further reduction in the proliferative potential and a shift towards differentiation. Among downregulated genes in both 13-HPODE-treated and LA-treated cells were *POLD2, MCM7*, and *PCNA*, which are involved in DNA replication; *CDC25A*, which is important for cell cycle progression; and *RPP40* and *EIF5A*, which are involved in RNA processing and translation. On the other hand, tumor suppressor genes including *APC, KLF4*, and *FOXO4* were upregulated in 13-HPODE-treated cells. In LA-treated cells, we observed downregulation of the proto-oncogene, *JUN*, and upregulation of the tumor suppressor genes *KLF7* and *FOXO4*. Interestingly, LA treatment caused a reduction in the expression of cell adhesion molecules *CEACAM1/M5/M6,* which are considered biomarkers of tumor progression [83]; this may also indicate reduced proliferation. LOOH-derived oxidative stress can cause base modification and DNA damage [47] due to the formation of DNA adducts via interaction between nucleotides and malondialdehyde (MDA), hence the protective response of DNA synthesis reduction was required to prevent further DNA damage by LOOH end-products [84]. Despite the various studies on LOOH-induced DNA damage, the mechanism by which LOOHs reduce DNA synthesis needs to be further investigated. 

In support of shifting towards differentiation, the focal adhesion pathway, which is involved in cell motility, differentiation, and other processes, was enhanced (*p*-value 0.06) in 13-HPODE-treated cells relative to untreated cells. Among the related induced genes was *COL7A1*. In the intestine, this pathway plays a role in epithelial barrier homeostasis and repair and tight junction organization [85]. This may indicate enhanced cellular differentiation, which promotes the development of brush borders, as evidenced by the upregulated *VIL1* gene in 13-HPODE-treated cells. In addition to the upregulation of *VIL1,* the sucrase-isomaltase gene, *SI*, which is another marker of intestinal differentiation [86] showed elevated expression in LA-treated cells compared to 13-HPODE-treated cells, indicating enhanced cellular differentiation. Furthermore, we observed upregulation of retinol metabolism in LA-treated cells, relative to untreated cells, which has been shown to play a role in intestinal epithelial cell processes including growth and differentiation [87]. In addition, *UGT2B4*, a detoxifying enzyme that plays a role in the retinol metabolic pathway, was detected and upregulated in LA-treated cells.

Our results showed a reduction in the oxidative phosphorylation pathway in both 13-HPODE- and LA-treated cells relative to untreated cells. Disrupted oxidative phosphorylation has been previously reported in mice fed with oxidized linoleic acid [88]. Moreover, mitochondrial components including membranes and matrix were among the top enriched GO terms in both 13-HPODE- and LA-treated cells, relative to untreated cells, which indicates the significant effect of 13-HPODE and LA on mitochondrial function. The *TOMM5* gene, which is a translocase of the outer mitochondrial membrane, was downregulated in both treatments. It has been reported that PUFAs can cause a change in mitochondrial membrane composition and organization that leads to increased production of ROS, which in turn causes peroxidation of membrane phospholipids and mitochondrial dysfunction [89].

Toll-like receptor (TLR) signaling was downregulated in LA-treated Caco-2 cells relative to untreated cells, which supports the previously reported inhibitory effect of PUFAs on TLR activation and inflammatory gene expression [90]. Specifically, n-3 PUFAs appeared to be more powerful inhibitors of TLR signaling than n-6 PUFAs [91]. There was a relative similarity in the data results between LA- and 13-HPODE-treated cells, suggesting the possibility of LA being oxidized in the intestinal epithelium, which has been previously reported [92] (Table 3).

## 5. Conclusions

The results of our transcriptomic profiling of Caco-2 cells carried out under standard in vitro conditions shed light on the response of intestinal epithelial cells to 13-HPODE or LA in terms of gene expression and pathway enrichment. The results presented in this study suggest that the most common dietary peroxidized lipid, 13-HPODE, may, on the one hand, enhance bile acid conjugation, which alters lipid uptake and might contribute to intestinal injury. On the other hand, 13-HPODE may affect multiple pathways of lipid metabolism including steroid hormone synthesis, which might affect intestinal barrier and immune function; and PPAR signaling, which alters fatty acid and glucose metabolism, energy production, and mitochondrial function leading to alteration of gut physiology. In addition, 13-HPODE as well as LA may have the ability to promote the antioxidant response and detoxification by cytochrome P450 in intestinal epithelial cells. Moreover, both treatments could reduce proliferative potential and could play a role in the absorptive cell differentiation and survival fates as they might suppress pathways involved in the cell cycle, DNA synthesis/repair, and enhance focal adhesion pathways. While similar effects could be seen in liver cells, it is conceivable that dietary peroxidized LA could have an impairment effect on energy production and lipid storage. In conclusion, this in vitro study using Caco-2 cells provides insights into the physiological changes that might occur in the intestinal epithelium upon exposure to 13-HPODE and the possible mechanisms by which it contributes to disease. Future directions of this research include studying the response of Caco-2 cells to LOOH using an experimental setting that simulates in vivo intestinal environment, such as the INFOGEST method [93].

## Figures and Tables

**Table 1:** Pathway enrichment analysis in 13-HPODE-treated Caco-2 cells. Pathway enrichment demonstrates upregulated and downregulated Kyoto Encyclopedia of Genes and Genomes (KEGG) pathways in 13-HPODE-treated Caco-2 differentiated cells relative to untreated cells.

KEGG Pathway (Upregulated)	*p*-Value
I00140 Steroid hormone biosynthesis	0.018
I04976 Bile secretion	0.029
I04974 Protein digestion and absorption	0.029
I00980 Metabolism of xenobiotics by cytochrome P450	0.062
I04510 Focal adhesion	0.069
I03320 PPAR signaling pathway	0.199
**KEGG Pathway (Downregulated)**	***p*-Value**
I03040—Spliceosome	0.0001
I00240—Pyrimidine metabolism	0.0004
I03008—Ribosome biogenesis in eukaryotes	0.0005
I03013—RNA transport	0.002
I00190—Oxidative phosphorylation	0.003
I04110—Cell cycle	0.004
I00230—Purine metabolism	0.006
I03420—Nucleotide excision repair	0.009
I03020—RNA polymerase	0.009
I03050—Proteasome	0.014
I03030—DNA replication	0.018
I04120—Ubiquitin-mediated proteolysis	0.030
I03410—Base excision repair	0.031
I03010—Ribosome	0.034

**Table 2:** Pathway enrichment analysis in LA-treated Caco-2 cells. Pathway enrichment demonstrates upregulated and downregulated KEGG pathways in LA-treated Caco-2 differentiated cells relative to untreated cells.

KEGG Pathway (Upregulated)	*p*-Value
I00830—Retinol metabolism	0.010
I04080—Neuroactive ligand-receptor interaction	0.017
I00980—Metabolism of xenobiotics by cytochrome P450	0.017
I00982—Drug metabolism—cytochrome P450	0.039
I04974—Protein digestion and absorption	0.047
**KEGG Pathway (Downregulated)**	***p-*Value**
I03008—Ribosome biogenesis in eukaryotes	0.0001
I03040—Spliceosome	0.0004
I03013—RNA transport	0.0007
I00240—Pyrimidine metabolism	0.0009
I00190—Oxidative phosphorylation	0.016
I03020—RNA polymerase	0.017
I03050—Proteasome	0.020
I00230—Purine metabolism	0.037
I04110—Cell cycle	0.038
I03420—Nucleotide excision repair	0.043
I04620—Toll-like receptor signaling pathway	0.045

**Table 3:** Comparative differential gene expression. Comparison of differential expression (↑ upregulation or ↓ downregulation) of selected genes in the two treatment cell groups relative to the untreated cell group.

Gene	13-HPODE-Treated	LA-Treated
*DKK1*	↓↓ ^3^	↓↓
*CPT1A*	↑↑ ^3^	↑ ^2^
*PLIN2*	↑↑	↑
*ODC1*	↓ ^2^	↓
*CREB3L3*	↑↑	↑
*FABP1*	↑↑	↑
*PDK4*	↑↑	↑
*PCK1*	↑↑	↑
*COL7A1*	↑↑	↑
*NOX1*	↓	↓
*AKR1C2*	↑↑	↑
*SOS1*	↑	↑
*BAAT*	↑	n ^1^
*CYP2B6*	↑↑	↑
*CYP2C9*	↑	↑
*INSIG1*	n	↓
*DDIT4*	↑	↑
*DUSP4*	↓	↓↓
*UGT2B4*	↑	↑
*TXNRD1*	↑	n
*HMOX1*	↑	n
*GSTP1*	↓	n
*CXCL1*	↓	↓
*GPX1*	↓	↓
*FOXO4*	↑	↑
*CEACAM6*	↓	↓↓
*POLD2*	↓	↓
*ADH6*	n	↑

^1^ n represents not differentially expressed (adjusted *p* > 0.05). ^2^ Single arrow (↑, ↓) represents differentially expressed (DE; adjusted *p* < 0.05). ^3^ Double arrows (↑↑, ↓↓) represent DE with more pronounced effect.

## Data Availability

All sequence files and metadata are available at NCBI under BioProject accession PRJNA682114. https://www.ncbi.nlm.nih.gov/bioproject/?term=PRJNA682114.

## Supplementary Materials

The following are available online at https://www.mdpi.com/2304-8158/10/2/314/s1, Figure S1: Library quality assessment. Complementary DNA library pool quality assessment using High Sensitivity D1000 ScreenTape assay showed good quality library with a maximum peak size of 337 bp. Figure S2. Principal component analysis (PCA). PCA plot visualizes sample-to-sample distances between untreated and 13-hydroperoxyoctadecadienoic acid (HPODE)-treated (**a**) or linoleic acid (LA)-treated (**b**) Caco-2 cells showing similarity with respect to treatment and good separation between untreated and treated groups. **Figure S3**: Log ratio vs. mean average (MA) plot. MA plots show DEGs (red dots; adjusted *p* < 0.05) between untreated Caco-2 cells and (**a**) 13-hydroperoxyoctadecadienoic acid (HPODE)-treated, or (**b**) linoleic acid (LA)-treated Caco-2 cells. *x*-axis represents the mean expression across untreated and treated cell groups; *y*-axis represents the log2 fold change between untreated and treated groups. Figure S4: Molecular function enrichment upon treating Caco-2 cells with 13-HPODE. Top enriched GO molecular functions in 13-HPODE-treated differentiated Caco-2 cells relative to untreated cells (adjusted *p* < 0.05). Figure S5: Cellular component enrichment upon treating Caco-2 cells with 13-HPODE. Top enriched GO cellular components in 13-HPODE-treated differentiated Caco-2 cells relative to untreated cells (adjusted *p* < 0.05). Figure S6: Molecular function enrichment upon treating Caco-2 cells with LA. Top enriched GO molecular functions in LA-treated differentiated Caco-2 cells relative to untreated cells (adjusted *p* < 0.05). Figure S7: Cellular component enrichment upon treating Caco-2 cells with LA. Top enriched GO cellular components in LA-treated differentiated Caco-2 cells relative to untreated cells (adjusted *p* < 0.05). Figure S8: Differential gene expression between 13-HPODE-treated and LA-treated cells. Heatmap shows the top 50 DEGs (adjusted *p* < 0.05) between 13-HPODE-treated Caco-2 cells (14D.H1, 14D.H2, and 14D.H3) and LA-treated cells (14D.LA1, 14D.LA2, and 14D.LA3). Green, upregulated; red, downregulated. Table S1: Primer sequences. Forward and reverse primer sequences used in quantitative RT-PCR. Table S2: Differential gene expression results of DESeq2 R package in 13-HPODE-treated cells. Differentially expressed genes (DEGs; adjusted *p* < 0.05) in 13-HPODE-treated Caco-2 cells (14D.H1, 14D.H2, and 14D.H3) compared to untreated control cells (14D.C1, 14D.C2, and 14D.C3). Table S3: Differential gene expression results of DESeq2 R package in LA-treated cells. DEGs (adjusted *p* < 0.05) in LA-treated Caco-2 cells (14D.LA1, 14D.LA2, and 14D.LA3) compared to untreated cells (14D.C1, 14D.C2, and 14D.C3). Table S4: Differential gene expression between 13-HPODE-treated and LA-treated cells. DEGs (adjusted p < 0.05) between 13-HPODE-treated Caco-2 cells (14D.H1, 14D.H2, and 14D.H3) and LA-treated Caco-2 cells (14D.LA1, 14D.LA2, and 14D.LA3).
