# Supplementary material for: Peroxidized Linoleic Acid, 13-HPODE, Alters Gene Expression Profile in Intestinal Epithelial Cells
Source: Foods. 2021 Feb 3;10(2):314. doi: 10.3390/foods10020314 (PMC7913489; doi:10.3390/foods10020314)
Supplement: Supplementary file 1 [file foods-10-00314-s001.zip › Supplementary Files/Supplementary Files-Figures.pdf]

Supplementary Figures:

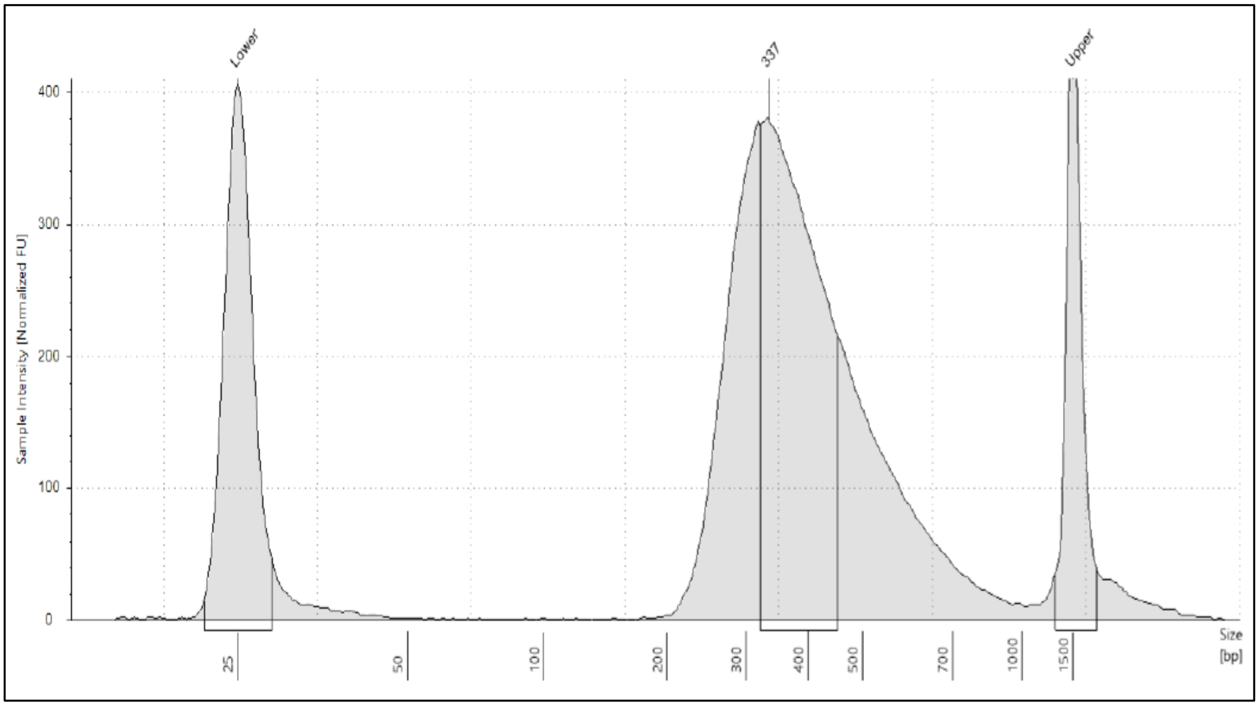

Figure S1. Library quality assessment. Complementary DNA library pool quality assessment using High Sensitivity D1000 ScreenTape assay showed good quality library with a maximum peak size of 337 bp.

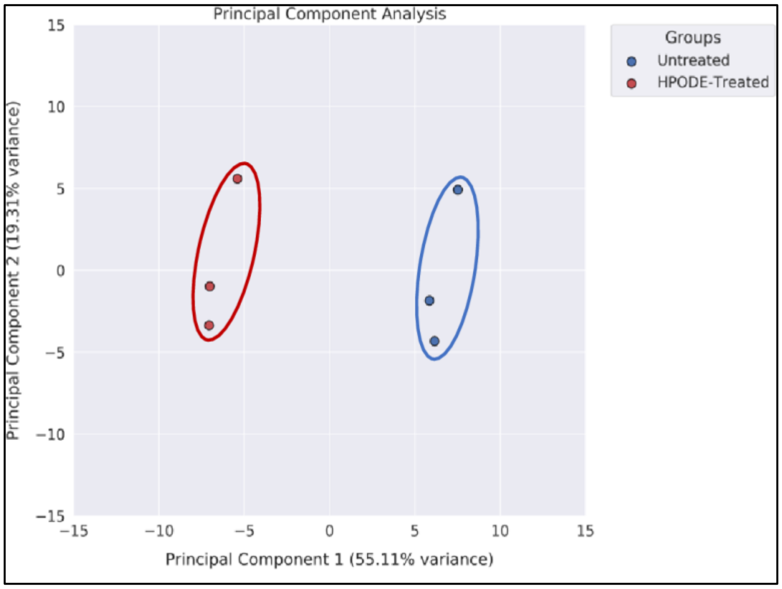

(a)

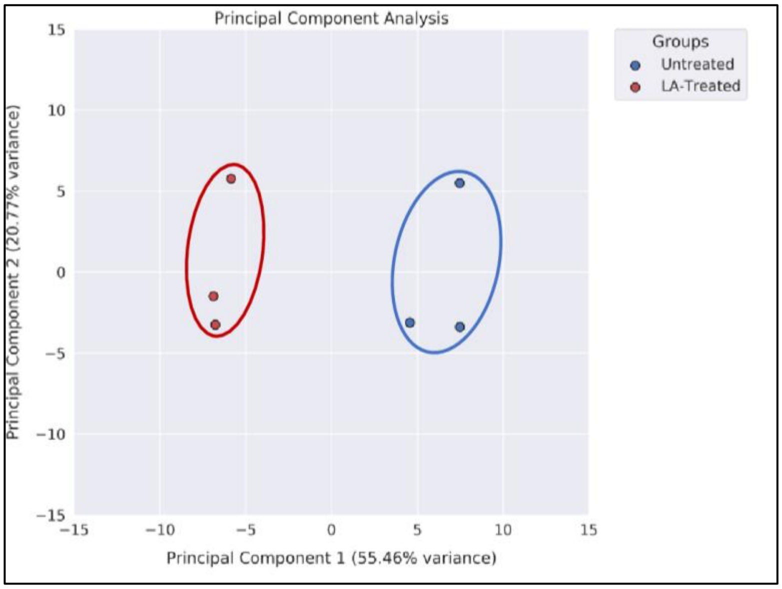

(b)

Figure S2. Principal component analysis (PCA). PCA plot visualizes sample-to-sample distances between untreated and 13-hydroperoxyoctadecadienoic acid (HPODE)-treated (a) or linoleic acid (LA)-treated (b) Caco-2 cells showing similarity with respect to treatment and good separation between untreated and treated groups.

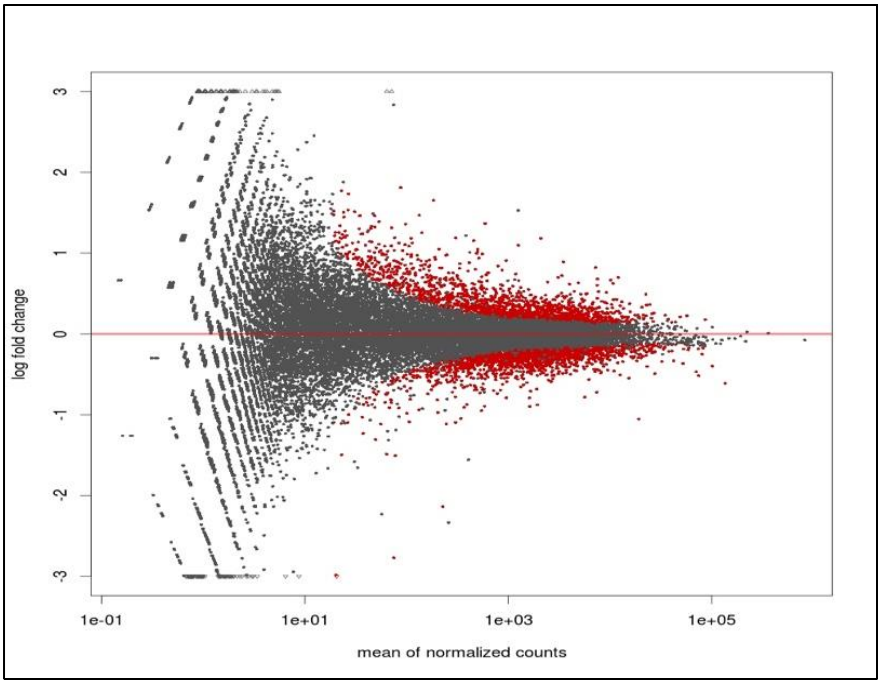

(a)

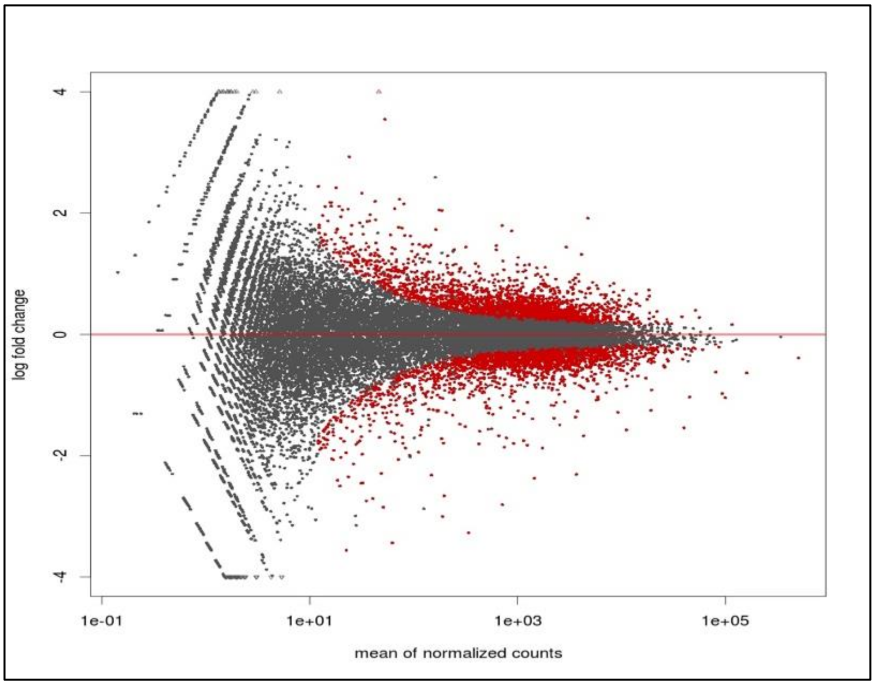

(b)

Figure S3. Log ratio vs. mean average (MA) plot. MA plots show DEGs (red dots; adjusted $p < 0.05$) between untreated Caco-2 cells and (a) 13-hydroperoxyoctadecadienoic acid (HPODE)-treated, (b) linoleic acid (LA)-treated Caco-2 cells. x-axis represents the mean expression across untreated and treated cell groups, y-axis represents the log2 fold change between untreated and treated groups.

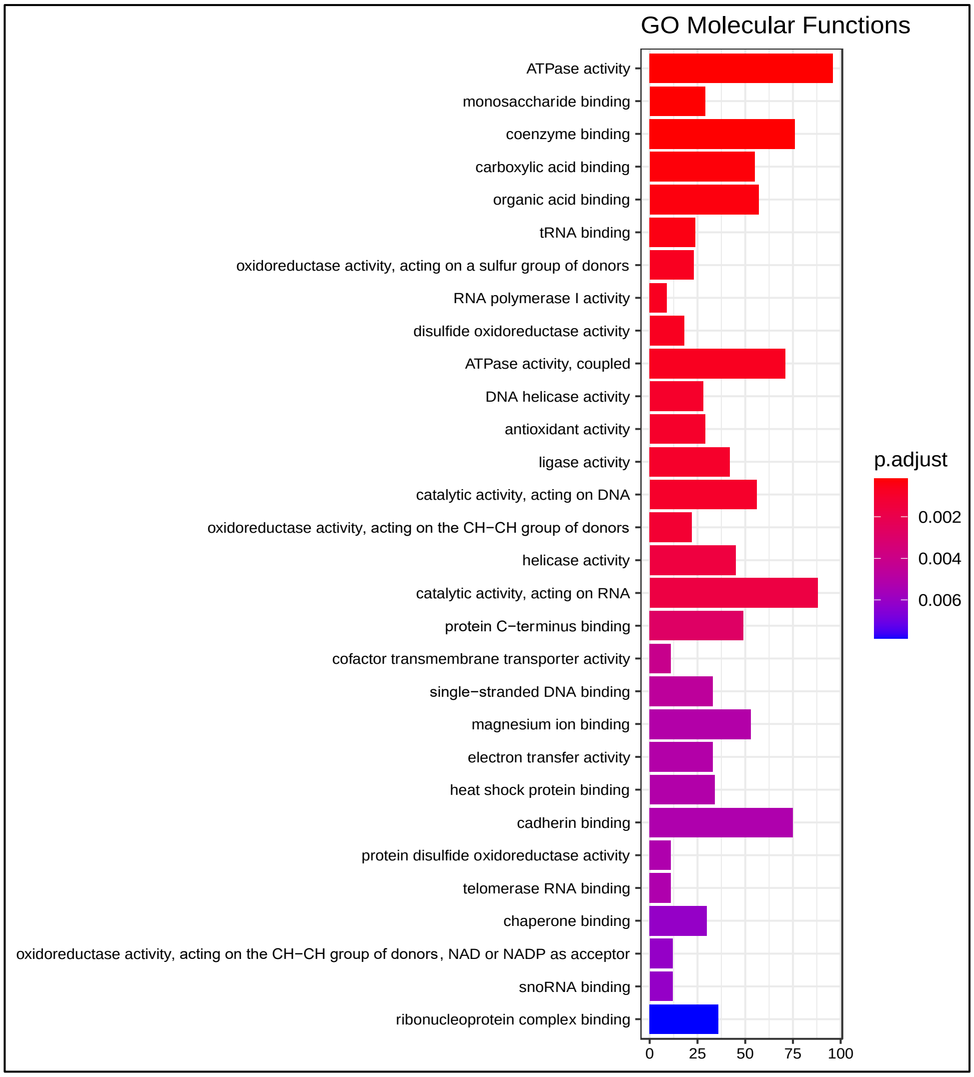

Figure S4. Molecular function enrichment upon treating Caco-2 cells with 13-HPODE. Top enriched Gene Ontology (GO) molecular functions in 13-HPODE-treated differentiated Caco-2 cells relative to untreated cells (adjusted $p < 0.05$).

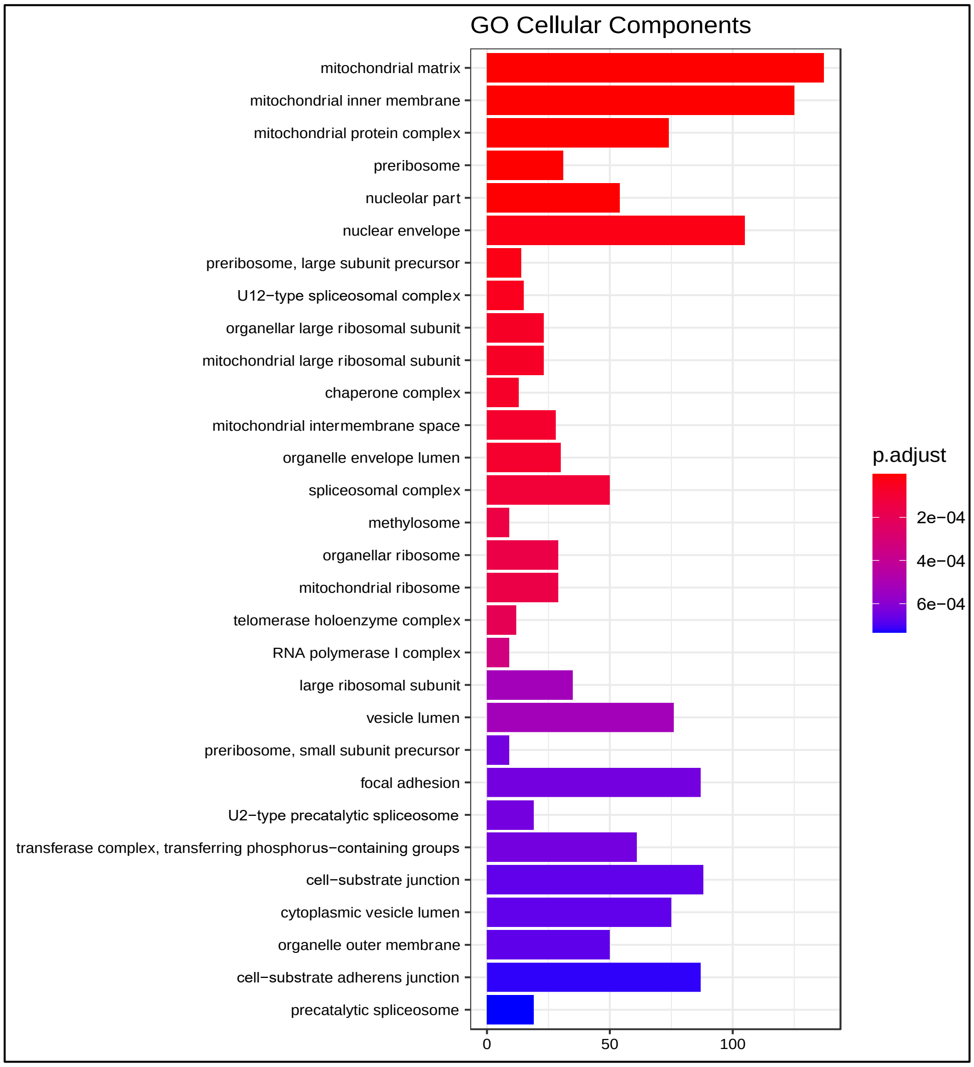

Figure S5. Cellular component enrichment upon treating Caco-2 cells with 13-HPODE. Top enriched GO cellular components in 13-HPODE-treated differentiated Caco-2 cells relative to untreated cells (adjusted $p < 0.05$).

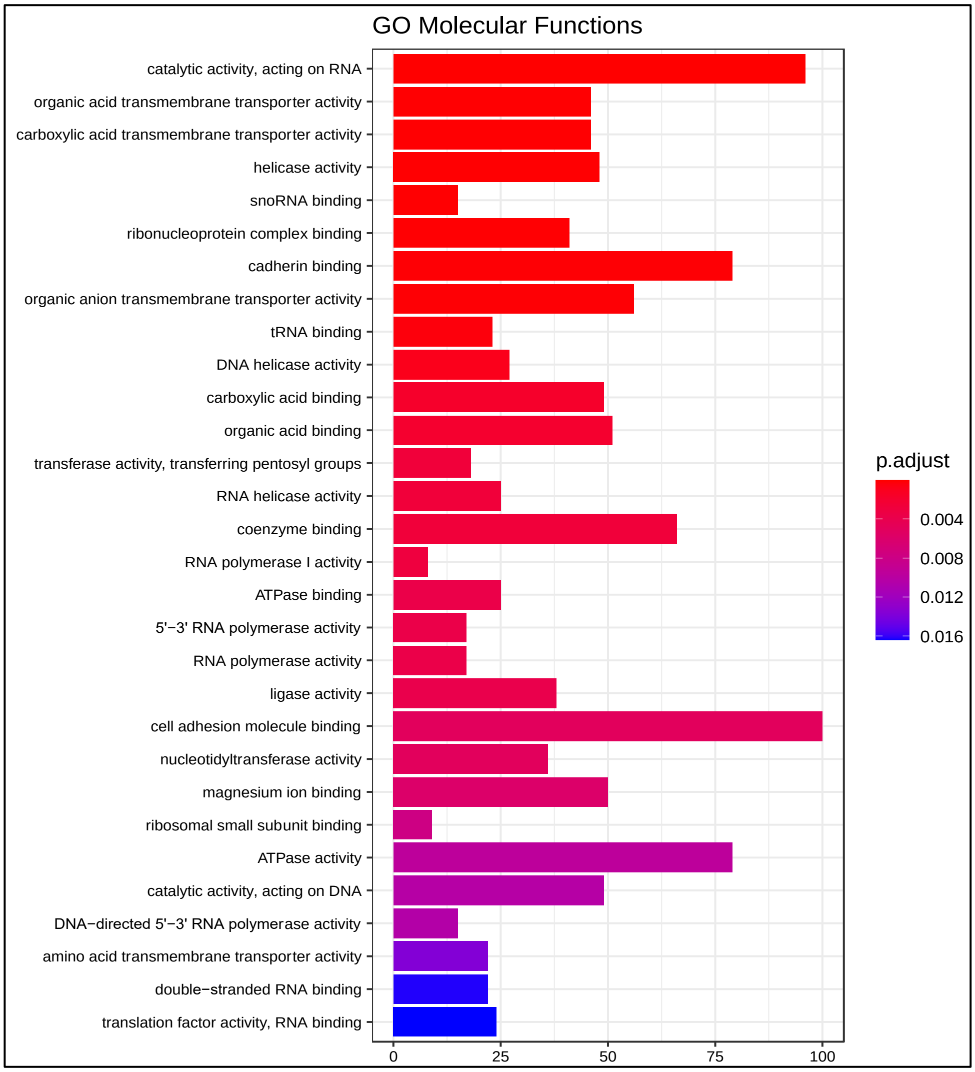

Figure S6. Molecular function enrichment upon treating Caco-2 cells with LA. Top enriched GO molecular functions in LA-treated differentiated Caco-2 cells relative to untreated cells (adjusted $p < 0.05$).

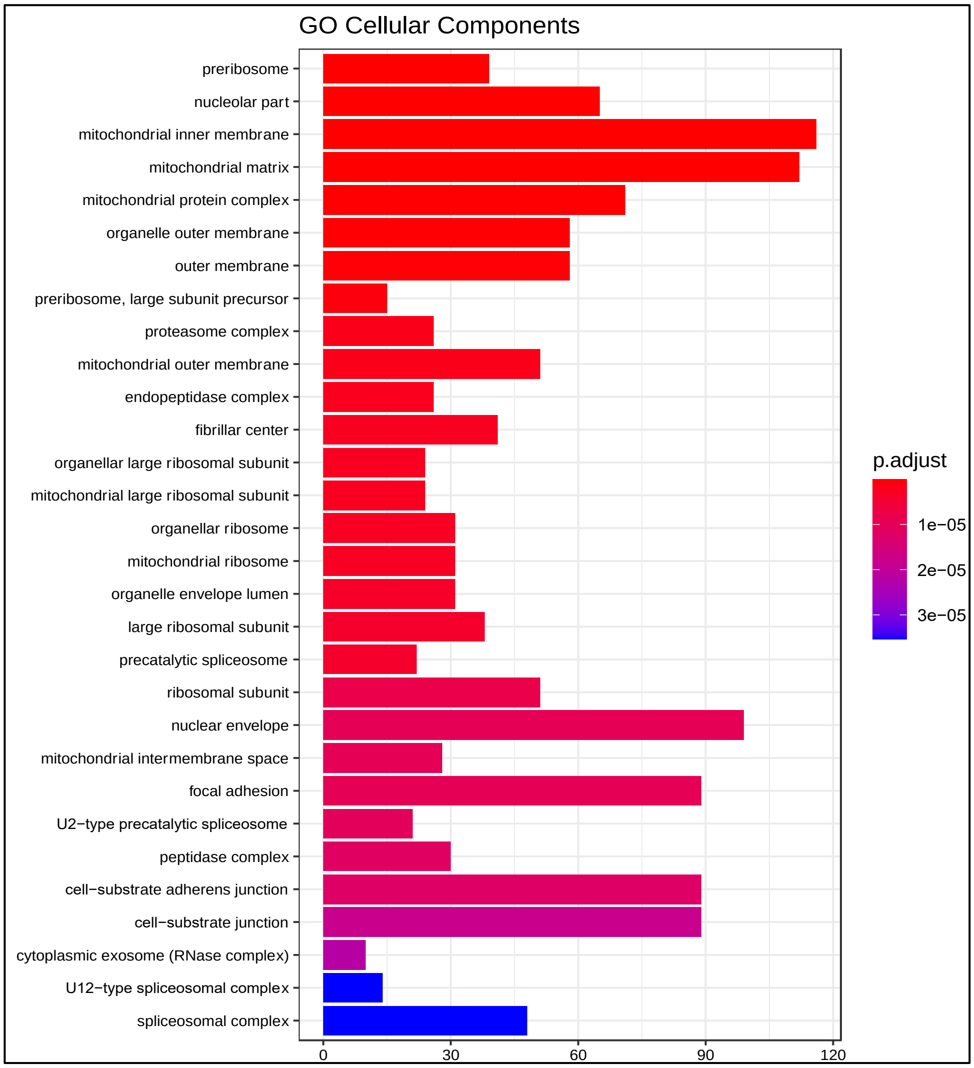

Figure S7. Cellular component enrichment upon treating Caco-2 cells with LA. Top enriched GO cellular components in LA-treated differentiated Caco-2 cells relative to untreated cells (adjusted $p < 0.05$).

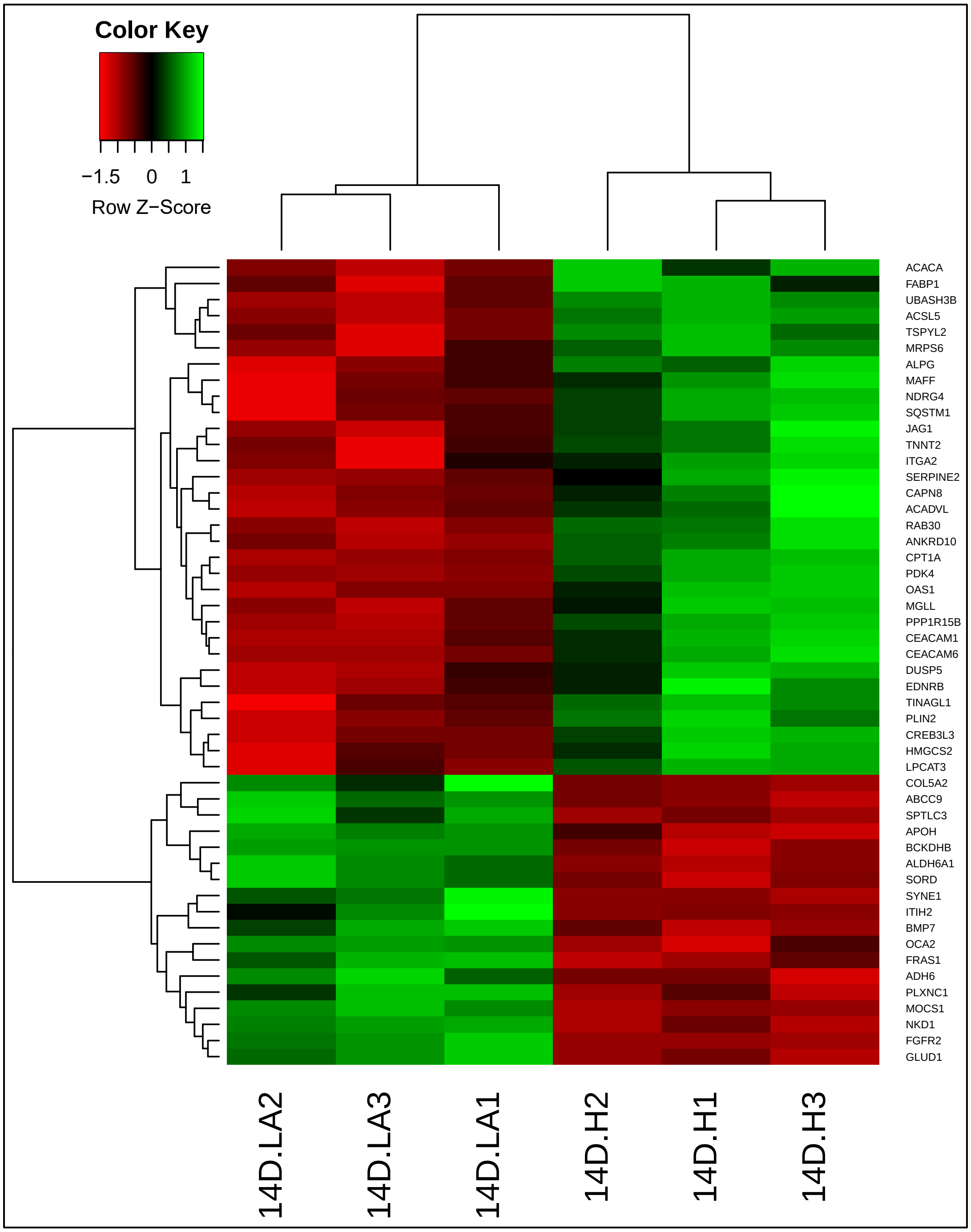

Figure S8. Differential gene expression between 13-HPODE-treated and LA-treated cells. Heatmap shows the top 50 DEGs (adjusted *p* < 0.05) between 13-HPODE-treated Caco-2 cells (14D.H1, 14D.H2 & 14D.H3) and LA-treated cells (14D.LA1, 14D.LA2 & 14D.LA3). (Green, upregulated; red, downregulated).
